# Critical Analysis of Stage IV Epithelial Ovarian Cancer Patients after Treatment with Neoadjuvant Chemotherapy followed by Cytoreductive Surgery and Hyperthermic Intraperitoneal Chemotherapy (CRS/HIPEC)

**DOI:** 10.1155/2020/1467403

**Published:** 2020-12-16

**Authors:** Carlos A. Munoz-Zuluaga, Armando Sardi, Michelle Sittig, Vadim Gushchin, Mary C. King, Carol Nieroda, Felipe Lopez-Ramirez, Teresa P. Diaz-Montes

**Affiliations:** ^1^Department of Surgical Oncology, The Institute for Cancer Care at Mercy Medical Center, Baltimore, Maryland 21202, USA; ^2^The Lya Segall Ovarian Cancer Institute at Mercy Medical Center, Baltimore, Maryland 21202, USA

## Abstract

**Background:**

Cytoreductive surgery and hyperthermic intraperitoneal chemotherapy (CRS/HIPEC) after neoadjuvant chemotherapy (NACT) showed promise as initial treatment for stage IIIC (SIII) epithelial ovarian cancer (EOC); however, stage IV (SIV) outcomes are rarely reported. We assessed our experience and outcomes treating newly diagnosed SIV EOC with NACT plus CRS/HIPEC compared to SIII patients.

**Methods:**

Advanced EOC from 2015–2018 managed with NACT (carboplatin/paclitaxel) due to unresectable disease or poor performance status followed by interval CRS/HIPEC were reviewed. Perioperative factors were assessed. Overall survival (OS) and progression-free survival (PFS) were analyzed by stage.

**Results:**

Twenty-seven FIGO stage IIIC (*n* = 12) and IV (*n* = 15) patients were reviewed. Median NACT cycles were 3 and 4, respectively. Post-NACT omental caking, ascites, and pleural effusions decreased/resolved in 91%, 91%, and 100% of SIII and 85%, 92%, and 71% of SIV. SIII/SIV median PCI was 21 and 20 obtaining 92% and 100% complete cytoreduction (≤0.25 cm), respectively. Median organ resections were 6 and 7, respectively. Grade III/IV surgical complications were 0% SIII and 23% SIV, without hospital mortality. Median time to adjuvant chemotherapy was 53 and 74 days, respectively (*p*=0.007). SIII OS at 1 and 2 years was 100% and 83% and 87% and 76% in SIV (*p*=0.269). SIII 1-year PFS was 54%; median PFS: 12 months. SIV 1- and 2- year PFS was 47% and 23%; median PFS: 12 months (*p*=0.944).

**Conclusion:**

Outcomes in select initially diagnosed and unresectable SIV EOC are similar to SIII after NACT plus CRS/HIPEC. SIV EOC may benefit from CRS/HIPEC, and further studies should explore this treatment approach.

## 1. Introduction

Epithelial ovarian, fallopian tube, and primary peritoneal cancers, known as epithelial ovarian cancer (EOC), are heterogeneous diseases staged and treated similarly [1, 2]. These diseases account for the majority of deaths from gynecological cancers in developed countries, due to scarcity of symptoms at early stages and lack of screening methods [3]. Consequently, most EOC patients are diagnosed after peritoneal spread (International Federation of Gynecology and Obstetrics (FIGO) stage III/IV) with 5-year survival of 29% [3].

Primary debulking surgery (PDS) followed by postoperative or adjuvant systemic chemotherapy (ASC) with taxane-platinum combinations is standard for advanced EOC (AEOC) [4]. However, over the past decade, new strategies have been pursued to improve outcomes, including the use of neoadjuvant systemic chemotherapy (NACT) and, more recently, intraoperative hyperthermic intraperitoneal chemotherapy (HIPEC) [5–10]. NACT plus interval cytoreductive surgery (CRS) without HIPEC demonstrated improved perioperative outcomes but nonsuperiority in terms of time to recurrence and survivals compared to PDS [5, 11, 12]. Recently, NACT plus interval CRS with HIPEC showed improved survival for stage III patients, but its' role in stage IV patients, who typically have limited treatment options and high mortality, is unclear [13, 14]. We assessed our experience treating newly diagnosed stage IV (SIV) EOC with NACT plus interval CRS/HIPEC and compared findings to the same treatment cohort of stage IIIC (SIII) patients.

## 2. Patients and Methods

An institutional CRS/HIPEC database was reviewed, identifying newly diagnosed AEOC patients who received NACT followed by CRS/HIPEC from 2015–2018. This treatment approach was offered at our institution to AEOC patients deemed ineligible for the randomized clinical trial (NCT 02124421) which assesses the role of CRS/HIPEC as initial treatment in AEOC. NACT criteria included unresectable peritoneal disease due to extensive small bowel or porta hepatis involvement, biliary obstruction, or encasement of common/external iliac vessels evidenced by imaging and/or laparoscopy, extra-abdominal disease, Eastern Cooperative Oncology Group (ECOG) performance status >2, and/or large volume of ascites or pleural effusion. Staging occurred before NACT and after CRS/HIPEC, assigning the highest staging for analysis.

Three cycles of NACT with systemic taxane/platinum regimens were administered. NACT response and surgical eligibility was evaluated with imaging, tumor markers, and performance status. Interval CRS/HIPEC was offered if complete CRS (residual disease <0.25 cm) was feasible, followed by 3 cycles of taxane/platinum ASC, totaling 6 cycles.

### 2.1. Response Evaluation

Treatment response was based on CT scan of chest/abdomen/pelvis pre-/post-NACT using Response Evaluation Criteria in Solid Tumors (RECIST) version 1.1 [15]. To note, the noncomplete response/nonprogressive disease (non-CR/non-PD) label is used over stable disease for patients with only nonmeasurable disease in this classification. [15] CA-125 levels were measured pre-NACT, post-NACT/pre-CRS/HIPEC, post-CRS/HIPEC, and post-ASC. Histopathologic chemotherapy response is not reported due to lack of consensus [16].

### 2.2. Interval Cytoreductive Surgery/Hyperthermic Intraperitoneal Chemotherapy (CRS/HIPEC) Procedure

Post-NACT, patients were considered CRS/HIPEC candidates if there was no gross extra-abdominal disease, gross resolution of prior pleural effusion, ECOG performance status ≤2, and complete cytoreduction was feasible (<0.25 cm residual disease). Intraoperative disease burden was estimated using Peritoneal Cancer Index (PCI) scores [17]. Multiple peritoneal and visceral resections were performed to reduce tumor to microscopic levels. Completeness of cytoreduction (CC) score quantified residual tumor with CC-0 (no visible residual disease) and CC-1 (residual tumor <0.25 cm) considered complete cytoreductions [18]. Incomplete cytoreduction was defined as tumor nodules 0.25 cm–2.5 cm (CC-2) or >2.5 cm (CC-3). HIPEC agents included carboplatin 800 mg/m^2^ (90 minutes, 41–43°C) or melphalan 50 mg/m^2^ (90 minutes, 41–42°C), if platinum resistance was suspected based on NACT response. Bowel anastomoses and chest tube placement were performed following perfusion. All procedures were performed together by an experienced team of gynecologic and surgical oncologists, specializing in peritoneal surface malignancies. Patients were transferred to the intensive care unit (ICU) for 24 hours and then to the inpatient oncology unit when clinically stable. Surgical complications were considered until postoperative day (POD) 90 and graded according to Clavien-Dindo classification with grade III/IV considered major [19].

### 2.3. Staging

AJCC 8^th^ edition staging was performed before NACT and at CRS/HIPEC using the highest staging for subgroup designation [20]. Stage IV disease included positive pleural effusion cytology (pM1a or FIGO stage IVA), liver or splenic parenchymal metastases, extra-abdominal metastases, including inguinal and extra-abdominal lymph nodes, and/or transmural intestinal involvement (cM1b/pM1b or FIGO stage IVB).

### 2.4. Follow-Up

Postoperative follow-up occurred 3 and 6 weeks after discharge, every 3 months for 2 years, and every 6 months thereafter, for 5 years. After ASC, CT scan of chest/abdomen/pelvis were performed every 6 months or, as clinically indicated. After complete cytoreduction (CC-0/1), disease recurrence was defined by radiographic/pathologic evidence or disease.

### 2.5. Statistical Analysis

Categorical variables were analyzed using chi-squared or Fisher's exact test and continuous variables using independent sample Student's *t* test or MannWhitney *U* test, when not normally distributed. Survival analysis was performed using KaplanMeier method and log-rank test. Overall survival (OS) was calculated from CRS/HIPEC to date of death. Progression-free survival (PFS) was calculated from CRS/HIPEC to date of radiographic/pathologic disease recurrence, or date of death from disease, whichever occurred first. PFS was only calculated with complete cytoreduction (CC-0/1). Median follow-up time was estimated using the reverse KaplanMeier method. All analyses were performed using STATA version 12.0 (StataCorp LLC, College Station, TX, USA) and considered statistically significant if *p* ≤ 0.05.

### 2.6. Ethics

IRB approval and preoperative consent were obtained.

## 3. Results

**Figure 1:**
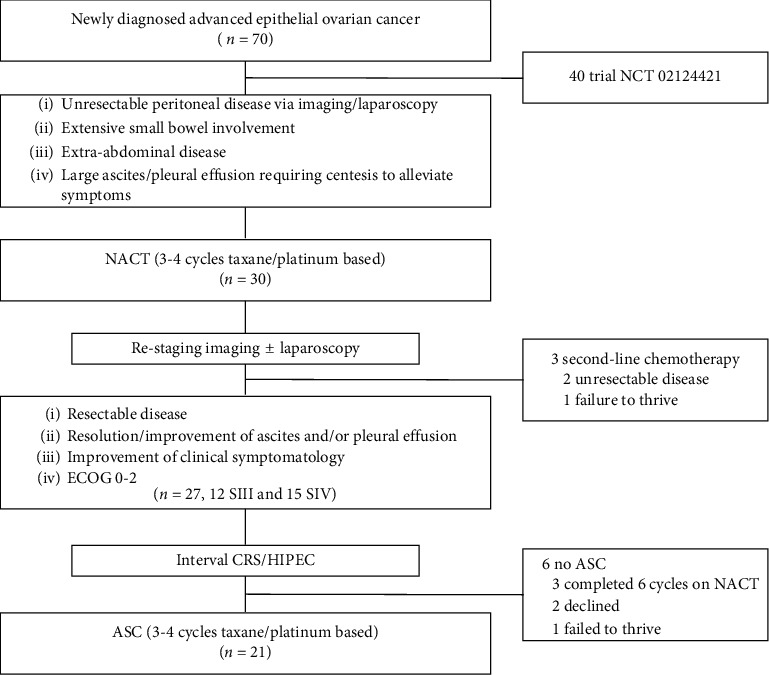
Management algorithm of initially unresectable stage IIIC and IV ovarian cancer patients. ASC: Adjuvant systemic chemotherapy, CRS/HIPEC: Cytoreductive surgery and hyperthermic intraperitoneal chemotherapy, ECOG: Eastern Cooperative Oncology Group Performance Status, NACT: Neoadjuvant systemic chemotherapy, SIII: Stage IIIC, SIV: Stage IVA/B.

Thirty AEOC patients received NACT from January 2015–December 2018. Three (10%) were not surgical candidates for CRS/HIPEC due to progression through NACT with unresectable disease (*n* = 2) and failure to thrive (*n* = 1). Two of these patients died 7.9 and 10.6 months from diagnosis and 4.9 and 8.6 months after completing NACT. The other patient was lost to follow-up with an unknown status. Twenty-seven patients received interval CRS/HIPEC: 12, stage IIIC (SIII) and 15, stage IVA/B (SIV) (Figure 1 and Table 1). Distant metastases in SIV included extra-abdominal metastases (inguinal and extra-abdominal lymph nodes) in 7 (47%), positive pleural effusion cytology in 5 (33%), transmural intestinal involvement in 2 (13%), and splenic parenchymal metastasis in 1 (7%) patient.

### 3.1. Response to Neoadjuvant Systemic Chemotherapy

According to RECIST 1.1 criteria, [15] 12 patients (SIII = 5/SIV = 7, 44%) had measurable disease (lymph nodes, masses) and 15 (SIII = 7/SIV = 8, 56%) had only nonmeasurable disease (mesenteric caking, effusions) after NACT. Complete response (CR) was seen in 4 (SIII = 1/SIV = 3, 15%), partial response in 4 (SIII = 2/SIV = 2, 15%), stable disease in 6 (SIII = 3/SIV = 3, 22%), non-CR/non-PD in 9 (SIII = 5/SIV = 4, 33%), and PD in 4 (SIII = 1/SIV = 3, 15%) patients. Patients with PD proceeded to CRS/HIPEC when there was evidence of reduction to bulky disease and complete cytoreduction was deemed feasible, despite evidence of new lesions after NACT.

Disease burden was also measured radiographically by extent of remaining omental disease, volume of ascites/pleural effusion, and lesion size pre/post-NACT. Reduction/resolution in each parameter was seen in 91%, 91%, 100%, and 67% SIII and 85%, 92%, 71%, and 92% SIV, respectively (Table 1).

### 3.2. Interval CRS/HIPEC Characteristics

Intraoperative characteristics are described in Table 2. Bowel anastomoses (colorectal, small bowel, ileocolonic, and colocolonic) were performed in 22 (81%) with 13 (48%) requiring 1 anastomosis (SIII = 5/SIV = 8) and 9 (33%) requiring 2 anastomoses (SIII = 4/SIV = 5). Ostomy creation was not required in any patient.

Complete cytoreduction (CC-0/CC-1) was achieved in 11 (92%) SIII and 15 (100%) SIV patients. Patients with CC-1 (*n* = 4, 15%, SIII = 3/SIV = 1) had residual nodules (<0.25 cm) in the mesentery, small bowel, or right upper quadrant. One SIII patient underwent incomplete cytoreduction (CC-2) with a sheath of scar tissue on the small bowel mesentery and distal ileum suspicious for disease that could not be completely excised.

HIPEC perfusion agents included carboplatin (*n* = 24, 89%, SIII = 12/SIV = 12) and melphalan (*n* = 3, 11%, SIV = 3). Median length of surgery was 8 hours in both groups. Median estimated blood loss was 425 mL SIII and 600 mL SIV (*p*=0.170). Intraoperative blood transfusions were required in 7 (58%) SIII and 9 (60%) SIV; median: 2 units (range: 1–3) in both groups. Postoperatively, 7 (58%) SIII and 14 (93%) SIV required transfusions (*p*=0.06); median: 2 units (range: 1–4). Median ICU stay was 1 day in both groups. Median hospital stay was 8 days in SIII and 11 days in SIV (*p*=0.01). All cases were high-grade serous carcinoma.

### 3.3. Postoperative Characteristics

Grade III/IV surgical complications occurred in 3 (20%) SIV, including reoperation for wound dehiscence (*n* = 1, POD 21), indwelling thorax catheter for pleural effusion (*n* = 1, POD 27), and pneumothorax (*n* = 1, POD 1) (Table 2). Postoperatively, all patients were anemic. Leukopenia was seen in 3 (25%) SIII and 7 SIV (47%) (*p*=0.42). Thrombocytopenia was seen in 7 (58%) SIII and 12 (80%) SIV (*p*=0.39). Twelve required granulocyte colony-stimulating factor agents (44%, SIII = 5/SIV = 7). Median CA-125 post-CRS/HIPEC was 17 U/mL in SIII and 13 U/mL in SIV patients. Median sampled lymph nodes were 20 (IQR: 3–29). Seventeen patients (65%, SIII = 7/SIV = 10) had positive lymph nodes.

Eleven (92%) SIII and 10 (67%) SIV received ASC, and median time to chemotherapy was 53 and 74 days, respectively (*p*=0.007). Median ASC was 3 cycles (range: 2–6). SIII received 2 (*n* = 2) and 3 (*n* = 9) cycles versus SIV who received 2 (*n* = 1), 3 (*n* = 7), and 4 (*n* = 2) cycles. Indications for no ASC were 6 NACT cycles requiring maintenance bevacizumab (*n* = 3), patient declined (*n* = 2), and failure to thrive (*n* = 1). Median CA-125 after ASC was 11 U/mL SIII and 9 U/mL SIV with abnormal levels noted in one SIII patient (40.6 U/mL) (Table 2).

### 3.4. Recurrence

Nine (81%) SIII and 12 (80%) SIV recurred after a median of 12 (IQR: 10.4–13) and 11 (IQR: 8.5–18.6) months, respectively. Disease recurrence limited to one region occurred in 12 patients: 5 abdominopelvic (SIII = 3/SIV = 2), 6 lymph nodes (SIII = 2/SIV = 4), and 1 distant site (nonabdominopelvic cavity/nonlymph node) (SIV = 1). Recurrence in multiple regions occurred in 10 patients: 3 abdominopelvic/lymph nodes (SIII = 2/SIV = 1), 2 abdominopelvic/distant site (SIII = 1/SIV = 1), 3 lymph node/distant site (SIII = 1/SIV = 2), and 2 abdominopelvic/lymph node/distant site (SIII = 1/SIV = 1). Sites of distant metastases were thorax (*n* = 6) and liver (*n* = 2) (Table 3).

Of 11 (92%) SIII ASC patients, 9 (82%) recurred after a median time of 9 months (IQR: 7–10) from the last ASC cycle and 12 months (IQR: 10–12) from CRS/HIPEC. One SIII without ASC recurred 8 months after CRS/HIPEC. Of 10 (67%) SIV ASC patients, 9 recurred after a median time of 11 months (IQR: 6–21) from the last ASC cycle and 16 months (IQR: 10–24) from CRS/HIPEC. Of 4 SIV patients without ASC, 3 recurred after 3, 7, and 9 months after CRS/HIPEC, and 1 died of another cause.

### 3.5. Survival and Progression-Free Survival

Overall survival at 1, 2, and 3 years was 92.6%, 79.4%, and 45.1%, respectively; median: OS 33 months. PFS at 1, 2, and 3 years was 50%, 21.2%, and 10.6%, respectively; median PFS: 11.8 months.

**Figure 2:**
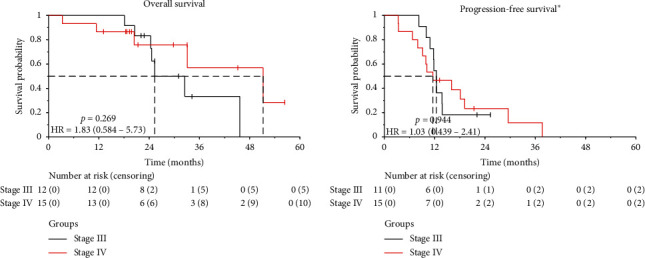
Overall survival and progression-free survival by disease stage in patients with peritoneal metastases from epithelial ovarian cancer treated with neoadjuvant systemic chemotherapy followed by cytoreductive surgery and hyperthermic intraperitoneal chemotherapy.^⋆^Progression-free survival was only calculated in patients with CC-0 or CC-1. HR: hazard ratio.

Five (42%) SIII and 10 (67%) SIV patients were alive after a median follow-up of 31 and 30 months, respectively. OS at 1, 2, and 3 years was 100%, 83.3%, and 33.3% in SIII and 86.7%, 75.8%, and 56.9% in SIV, respectively. Median OS was 25 and 51 months in SIII and SIV, respectively (*p*=0.269). One and 2 years PFS was 54.4% and 18.2% in SIII and 46.7% and 23.3% in SIV, respectively. Median PFS was 12.4 and 11.5 months in SIII and SIV, respectively (*p*=0.944) (Figure 2).

## 4. Discussion

Newly diagnosed FIGO SIV ovarian cancer patients who initially present with extensive, unresectable disease, or poor performance status are typically managed with a palliative approach. However, NACT followed by interval CRS/HIPEC can offer therapeutic benefit for these advanced patients. In our study, SIV had similar outcomes to and a tendency for improved cytoreduction rates (CC-0: 67% vs. 93%) and survival (median OS: 25 vs. 51 months) than SIII.

NACT prior to surgical debulking is considered under the premise that reducing tumor burden will improve patient performance and increase complete cytoreduction rates, resulting in lower morbidity, shorter hospital stay, and improved quality of life [10, 21–23]. However, no randomized trial has demonstrated superior survival of NACT plus interval debulking to primary debulking surgery (Table 4) [7–11, 24]. Moreover, the dominant factor influencing AEOC survival is the quality of cytoreduction, with >2 cm residual disease providing no survival benefit [4, 25–28]. Thus, NACT plus interval debulking has been reserved only for patients who are not surgical candidates, commonly due to pleural disease, massive ascites, or extensive small bowel involvement. Interestingly, despite the controversial benefit of NACT in a broad AEOC patient cohort, pooled analysis demonstrates that NACT offers better survival in SIV disease with high tumor burden or poor performance status [5]. In our study, 90% (27/30) who underwent NACT became surgical candidates achieving 96% complete cytoreduction rate and 9-day median hospital stay with encouraging cytoreduction rates and survival outcomes experienced in 15 SIV patients compared to SIII.

Traditionally, NACT is combined with standard debulking surgery; however, the addition of HIPEC during interval debulking surgery is gaining interest [6, 29, 30]. HIPEC has promising results treating other cancers that commonly present with peritoneal spread [31–33]. HIPEC allows for direct contact of high-dose, locoregional chemotherapy, potentiated by heat increasing cytotoxicity and tissue penetration, inhibiting angiogenesis, and inducting apoptosis [34–37]. Even after complete removal of macroscopic disease, microscopic tumor cells likely remain and could contribute to early recurrence rates [38]. This may especially be true after NACT where both complete and partial response to therapy can appear as scar tissue intraoperatively [39]. Therefore, the addition of HIPEC may offer improved outcomes for these patients.

Although taxane and platinum agents are commonly used with remarkable hyperthermic effects, further investigation into the optimal HIPEC agents is needed, especially in platinum/chemo-resistant disease [40, 41]. The majority of our patients (88%) received carboplatin. However, those with seemingly platinum-resistant disease requiring 6 NACT cycles (*n* = 3) were given melphalan, an alternative HIPEC agent for aggressive and recurrent peritoneal malignancies [42, 43].

Previous studies report the benefits of HIPEC for recurrent AEOC. Spiliotis et al. conducted the first prospective, randomized phase III study investigating HIPEC as an alternative treatment in recurrent stage IIIC/IV AEOC after PDS and systemic chemotherapy [44]. Patients underwent CRS/HIPEC (*n* = 60) or CRS alone (*n* = 60), both followed by ASC. PCI was ≥10 in 48% and 50% and CC-0 was achieved in 65% and 55%, by treatment group, respectively, with the HIPEC having significantly longer OS (median OS: 27 versus 13 months (*p*=0.006)). Despite the fact that these results are controversial, subanalysis by stage was not performed, and our cohort relates only to initially diagnosed patients; data from this first trial suggests that HIPEC may be beneficial in recurrent SIV AEOC [45].

Van Driel et al. published the results of their randomized phase III trial in SIII EOC comparing NACT and CRS/HIPEC (*n* = 122) vs. CRS alone (*n* = 123), both followed by ASC [6]. Disease burden was measured by the number of abdominal regions involved, rather than PCI, making comparisons to other HIPEC studies inequitable [6, 44]. Nevertheless, in HIPEC versus no-HIPEC groups, 6–8 regions were involved in 32% and 33%, with CC-0 achieved in 69% and 67%, respectively. Median OS was 46 versus 34 months (*p*=0.02), respectively, without significant differences in postoperative complications or health-related quality of life. Comparatively, our study included only initially unresectable patients including both SIII and SIV disease. Disease burden was extensive in our SIV patients (median PCI: 20) and CC-0 achieved in 93%, with median OS of 51 months.

Despite variations of reported OS, HIPEC consistently demonstrated improved survival compared to controls (Table 4) [6, 29, 44]. Variances could be explained by differences in disease burden, complete cytoreduction rates, or quality of surgery. Spiliotis et al. reported a shorter median OS (27 months) for recurrent SIII/IV compared to Van Driel et al. who reported 46 months median OS with NACT and interval CRS/HIPEC in SIII patients. However, the Spiliotis et al. study had higher disease burden and lower cytoreduction rates [44]. In our cohort, SIII and SIV intraoperative median PCI was 21 and 20, CC-0 was achieved in 67% and 93%, and median OS was 25 months and 51 months, respectively. Our population presented with high disease burden, even after NACT, and this may represent aggressive tumor biology although maximum surgical effort and high complete cytoreduction rates were achieved through combined surgical efforts of gynecologic and surgical oncologists. Regardless, SIV NACT and interval CRS/HIPEC patients in our cohort demonstrated comparable outcomes to unresectable SIII.

The absence of ostomies in our cohort highlights the collaboration between gynecologic and surgical oncologists. This can be compared to the 72% ostomy rate in the Van Driel et al. trials' CRS/HIPEC group and among other reports ranging from 17–97% after AEOC debulking surgery [6, 46, 47]. We were able to avoid ostomy creation despite single and double bowel anastomosis in 48% and 33%, respectively. Surgeons should be cognizant that performing bowel resections during CRS/HIPEC does not necessitate ostomy creation. Surgical oncologists are highly skilled and experienced in performing bowel resections with anastomoses. This enhances the collaborative effort between gynecologic and surgical oncology, translating to improved surgical outcomes and patient quality of life.

In our study, overall time from CRS/HIPEC to ASC was significantly longer compared to Van Driel trial (median time of 57 days versus 33 days, respectively). Nevertheless, median length of surgery was considerably longer in our cohort (8 versus 5.6 hours) suggesting extensive resections to achieve complete cytoreductions in patients with extensive disease. Disease burden and quality of cytoreduction play a role in the delay of ASC after CRS/HIPEC due to longer recovery time with extensive procedures and multiple organ resections.

We also found that median hospital stay and time to ASC were significantly longer in SIV than in SIII. Review of cases revealed that more than half of SIV patients had a hospital stay ≥10 days (60% SIV vs. 8% SIII) with median PCI of 24 when hospital stay exceeded 10 days vs. median PCI of 19 when hospital stay was <10 days (*p*=0.19). Furthermore, these SIV patients also had the longest time to ASC (>8 weeks) of which delays to adjuvant therapy were often patient driven. Thus, it seems that SIV patients with high PCI may require longer hospital stay and more time recovering before resuming chemotherapy.

Study limitations include its retrospective design at a single institution, small sample size, and limited follow-up. However, it includes detailed perioperative characteristics and outcomes of SIV disease, a subgroup that has shown to benefit the most from multimodal treatment approaches, such as NACT plus interval debulking surgery and warrant further studies [5]. SIV patients were excluded from the Van Driel et al. trial; nonetheless, these results and other reports are encouraging to further explore the role of NACT and interval CRS/HIPEC in SIV disease [6, 14, 29].

## 5. Conclusions

Promising survival outcomes, similar to stage IIIC, can be achieved for patients with initially unresectable, stage IV EOC treated with NACT and interval CRS/HIPEC. Randomized studies are needed to assess the long-term outcomes of NACT plus CRS/HIPEC in stage IV ovarian cancer.

## Figures and Tables

**Table 1:** Characteristics of epithelial ovarian cancer patients with neoadjuvant chemotherapy.

Characteristic	All population (*n* = 27)	Stage III (*n* = 12)	Stage IV (*n* = 15)	*p* value
Pre-NACT				
Median age at diagnosis [IQR], years	65 [59–70]	64 [58–65]	66 [62–74]	0.249
Median body mass index [IQR], kg/m^2^	26 [23.4–29.7]	28.2 [22–32.7]	25.9 [23.4–28.6]	0.354
Germline *BRCA*1/2 mutation, *n* (%)	1/19 (5)	1/8 (13)	0/11 (0)	—
Procedures with (+) cytology, *n* (%)				
Paracentesis	15/15 (100)	10/10 (100)	5/5 (100)	—
Thoracentesis	6/6 (100)	0 (0)	6/6 (100)	—
Median volume of paracentesis [IQR], mL	3000 [1600–5600]	3500 [1500–6025]	2600 [1450–4550]	—
Median volume of thoracentesis [IQR], mL	1500 [950–1700]	NA	1500 [950–1700]	—

NACT^*∗*^				
Median time from diagnosis to NACT [IQR], days	26 [19–33]	29 [20–34]	25 [15–36]	0.680
Median NACT cycles, (range)	3 (3–6)	3 (3–4)	4 (3–6)	0.080
Cycles of NACT, *n* (%)				
3 cycles	16 (59)	9 (75)	7 (47)	—
4 cycles	7 (26)	3 (25)	4 (27)	—
6 cycles†	4 (15)	0 (0)	4 (27)	—

NACT response				
Hypoalbuminemia‡, *n* (%)				
Before NACT	10 (37)	5 (41.7)	5 (33.3)	0.656
After NACT	1 (4)	0 (0.0)	1 (6.7)	1.000
Median CA-125 [IQR], U/mL				
Before NACT	1077 [500–3317]	993 [297–3124]	1168 [707–3460]	0.661
After NACT§	20.4 [11–69]	21 [12–68]	19 [8–70]	0.464
Radiographic response to NACT, *n* (%)				
Reduction or resolved, omental caking	21/24 (88)	10/11 (91)	11/13 (85)	—
Reduction or resolved, ascites	21/23 (91)	10/11 (91)	11/12 (92)	—
Reduction or resolved, pleural effusion	9/11 (82)	4/4 (100)	5/7 (71)	—
Reduction or resolved, lesions	18/21 (86)	6/9 (67)	12/13 (92)	—

^*∗*^All patients received taxane/platinum chemotherapy with bevacizumab added in 1 patient. †Four SIV patients received 6 cycles due to physician preference (*n* = 3) and coronary artery stent placement postponing surgery (*n* = 1). ‡Serum albumin levels <3.5 g/dL. §Five (42%) SIII and 6 (40%) SIV had abnormal CA-125 levels (>35 U/mL) Post-NACT (range = 36–228 U/mL). IQR: interquartile range; NACT: neoadjuvant chemotherapy.

**Table 2:** CRS/HIPEC and postoperative characteristics by stage.

Characteristic	Total population (*n* = 27)	Stage III (*n* = 12)	Stage IV (*n* = 15)	*p* value
CRS/HIPEC characteristics				
Median time from NACT to CRS/HIPEC [IQR], days	40 [35–45]	41 [30–47]	40 [35–45]	0.757
Median PCI [IQR]	20 [12–27]	21 [10–29]	20 [13–26]	0.926
Extent of resections, median [IQR]				
Organs resected	7 [5–8]	6 [5–8]	7 [6–8]	0.490
Visceral peritonectomies	2 [2-3]	2 [1–3]	2 [2-3]	0.409
Parietal peritonectomies	4 [3-4]	4 [3-4]	4 [3–5]	0.504
Pelvic mass, *n* (%)	24 (89)	9 (75)	15 (100)	0.075
Bowel anastomosis, *n* (%)				
0	5 (19)	3 (25)	2 (13)	—
1	13 (48)	5 (42)	8 (53)	—
2	9 (33)	4 (33)	5 (33)	—
CC score, *n* (%)				
CC-0 (no visible tumor)	22 (81)	8 (67)	14 (93)	—
CC-1 (tumor ≤0.25 cm)	4 (15)	3 (25)	1 (7)	—
CC-2 (tumor 0.25 cm–2.5 cm)	1 (4)	1 (8)	0 (0)	—
Median length of surgery [IQR], hours	8 [7–8]	8.3 [6.4–9.3]	8 [7–8.3]	0.482
Estimated blood loss [IQR], mL	500 [350–800]	425 [275–600]	600 [350–1000]	0.170
Transfusions, *n* (%)				
Intraoperative	16 (59)	7 (58)	9 (60)	1.000
Postoperative	21 (78)	7 (58)	14 (93)	0.060
Median length of hospitalization [IQR], days	9 [7–11]	8 [6–9]	11 [8–12]	**0.010**
Tumor site, *n* (%)				
Ovary	9 (33)	4 (33)	5 (33)	—
Fallopian tube	11 (41)	4 (33)	7 (47)	—
Primary peritoneal	7 (26)	4 (33)	3 (20)	—

Postoperative characteristics				
Grade III/IV surgical complications, *n* (%)	3 (11)	0 (0)	3 (23)	—
Hematologic toxicity, *n* (%)				
Anemia				
Preoperative	22 (82)	10 (83)	11 (73)	0.662
Postoperative	27 (100)	12 (100)	15 (100)	—
Leukopenia				
Preoperative	10 (37)	6 (50)	4 (27)	0.257
Postoperative	10 (37)	3 (25)	7 (47)	0.424
Thrombocytopenia				
Preoperative	1 (4)	0 (0)	1 (7)	1.000
Postoperative	19 (70)	7 (58)	12 (80)	0.398
Median CA-125 post-CRS/HIPEC [IQR] (*n*), U/mL	13 [8.2–23.2]	17 [8.2–28.6] (10)	13 [7.3–21.9] (12)	0.767
Positive lymph nodes, *n* (%)	17/26 (65)	7/11 (63)	10 (67)	0.706
Patients with ASC, *n* (%)	21 (78)	11 (92)	10 (67)	0.182
Median time from CRS/HIPEC to ASC [IQR], days	57 [51–73]	53 [40–58]	74 [56–91]	**0.007**
Median CA-125 post-ASC [IQR] (*n*), U/mL	9.5 [7–13]	11 [7–18.2] (11)	9 [7.3–12.3] (8)	0.385
Recurrence, *n* (%)	21 (81)	9/11 (81)	12 (80)	0.236
Alive, *n* (%)	15 (55)	5 (42)	10 (67)	—
Median follow-up (95% CI), months	31 [22–40]	31 [23–39]	30 [14–46]	—

ASC: adjuvant systemic chemotherapy, CC: completeness of cytoreduction score, CI: confidence interval, CRS/HIPEC: cytoreductive surgery and hyperthermic intraperitoneal chemotherapy, IQR: interquartile range, NACT: neoadjuvant chemotherapy.

**Table 3:** Relevant study population characteristics.

Patient ID	Primary tumor site	Highest FIGO stage	Staging^*a*^		No. of NACT cycles	NACT response^*b*^	No. of ASC cycles	Recurrence			Disease status	Time to recurrence (months)	Overall follow-up (months)
			Clinical (cTNM)	Post-NACT and CRS/HIPEC (ypTNM)				Intracavitary	Lymph nodes	Distant			
1	OV	IIIC	cT3c cN0 cM0	ypT3c ypN1 cM0	4	PD	2	—	R-iliac LN	—	DOD	13.75	24.61
2	PP	IVB	cT3c cN1 cM1b	ypT3c ypN1 cM1b	6	PD	NA	—	—	—	DOC	—	3.29
3	OV	IVA	cT3c cN1 pM1a	ypT3c ypN0 pM1a	3	Non-CR/non-PD	3	—	Portacaval LN	—	AWD	37.66	56.35
4	PP	IVA	cT3c cN1 pM1a	ypT3c ypN1 pM1a	4	Non-CR/non-PD	2	Loculated ascites, L-abdomen thickening	—	—	DOD	15.99	51.18
5	OV	IVA	cT3c cN0 pM1a	ypT3c ypN0 pM1a	3	PD	3	Pancreatic and L-abdomen implants	—	R-major and minor lung fissures thickening	DOD	7.93	20.43
6	OV	IIIC	cT3c cN0 cM0	ypT3b ypN0 cM0	3	Non-CR/non-PD	3	Mass adjacent to spleen, L-paramedian mass, L-external iliac nodule	—	—	DOD	12.43	45.63
7	OV	IVB	cT3c cN0 pM1a	ypT3c ypN1a pM1b	4	SD	4	Mesenteric and anterior abdominal wall nodules	L-external iliac LN	Hepatic metastases	AWD	18.13	45.16
8	PP	IIIC	cT3c cN0 cM0	ypT3c ypN0 cM0	3	PR	3	—	Para-aortic LN	Internal mammary LN	DOD	12.40	32.47
9	FT	IIIC	cT3c cN0 cM0	ypT3c ypN1 cM0	3	PR	3	Ascites and small peritoneal nodules	—	—	DOD	11.78	18.13
10	PP	IIIC	cT3c cN1 cM0	ypT0 ypN1a cM0	3	Non-CR/non-PD	3	Bilateral pelvic sidewall adenopathy	R-iliac and presacral LNs, L-para-aortic and retrocaval LNs	—	DOD	11.71	25.33
11	FT	IIIC	cT3c cN0 cM0	ypT3c ypN1a cM0	3	Non-CR/non-PD	3	Ascites and mesenteric enhancement	—	—	DOD	9.97	24.38
12	FT	IIIC	cT3c cN0 cM0	ypT3c ypN1a cM0	3	Non-CR/non-PD	3	—	Aortocaval, anterior aortic, L-para-aortic and aortic bifurcation LNs	—	AWD	10.86	34.24
13	FT	IVB	cT3c cN0 cM0	ypT3b ypN0 pM1b	3	CR	3	—	L-pelvic LN, LLQ and mid abdomen LNs	—	NED	19.14	33.06
14	FT	IVB	cT3c cN0 cM0	ypT3c ypN1a pM1b	4	PD	NA	—	—	R-pleural effusion, R-cardiophrenic and epicardial LN	DOD	6.71	33.09
15	FT	IIIC	cT3c cN0 cM0	ypT3c ypN0 cM0	3	SD	3	LUQ peritoneal nodule	—	L-epicardial LN	AWD	13.78	31.02
16	PP	IIIC	cT3c cN1 cM0	ypT3c ypN1 cM0	3	SD	NA	Presacral and perirectal densities, porta hepatis thickening, descending and sigmoid colon implants	R-pelvic LN, aortic bifurcation and anterior R-iliac LNs	Two small liver lesions	DOD	8.22	20.53
17	PP	IVB	cT3c cN0 cM1b	ypT3a ypN0 cM1b	4	PR	4	—	LN anterior to the 2^nd^ portion of the dueodenum	—	NED	29.54	29.80
18	PP	IIIC	cT3c cN1 cM0	ypT3a ypN0 cM0	4	Non-CR/non-PD	3	—	—	—	NED	—	25.30
19	FT	IVB	cT3c cN0 cM0	ypT3c ypN1 pM1b	3	Non-CR/non-PD	3	—	—	—	NED	—	21.35
20	OV	IIIC	cT3c cN1 cM0	ypT3c ypN1a cM0	3	SD	3	Cecal thickening	Posterior aortic and aortocaval LNs, R-pelvic LN	—	AWD	9.57	22.83
21	OV	IIIC	cT3c cN0 cM0	ypT0 ypN0 cM0	4	CR	2	—	—	—	NED	—	22.07
22	OV	IVB	cT3c cN1 cM1b	ypT3c ypN1b pM1a	3	SD	3	—	Para-aortic and aortocaval LNs	L-supraclavicular LN	AWD	10.10	19.80
23	FT	IVB	cT3c cN1 cM0	ypT3c ypN0 pM1b	3	SD	3	Cecal and anterior pancreatic nodules	—	—	AWD	9.87	18.68
24	OV	IVB	cT3c cN1 cM1b	ypT2a ypN1a cM1b	3	PR	3	LUQ peritoneal nodules	L-iliac LN	—	AWD	11.55	18.39
25	FT	IVA	cT3c cN0 pM1a	ypT3c ypN1a pM1a	6	Non-CR/non-PD	NA	—	—	—	NED	—	13.13
26	FT	IVB	cT3c cN1 cM1b	ypT2b ypN0 pM1a	6	CR	NA	—	R-inguinal, R-para-aortic and multiple bilateral retroperitoneal LNs	L-supraclavicular, R-paratracheal, R-axillary, pretracheal and R-prehilar LNs	DOD	3.36	11.41
27	FT	IVB	cT3c cN1 pM1b	ypT3a ypN0 pM1b	6	CR	NA	—	Multiples retroperitoneal and pelvic LN	—	AWD	9.11	19.31

^a^According to the 8th edition of the American Joint Committee on Cancer TNM staging system, and ^b^according to the Response Evaluation Criteria in Solid Tumors (RECIST) version 1.1; AWD: alive with disease, CC-Score: completeness of cytoreduction score, CR: complete response, DOC: dead of other cause, DOD: dead of disease, FT: fallopian tube, L: left, LNs: lymph nodes, LUQ: left upper quadrant, NA: not applicable, NACT: neoadjuvant systemic chemotherapy, NED: no evidence of disease, OV: ovarian, PD: progressive disease, PP: primary peritoneal, PR: partial response, R: right, and SD: stable disease

**Table 4:** Published randomized controlled trials assessing the benefit of NACT and/or HIPEC versus debulking surgery in advanced ovarian cancer.

Author and study type	Population and Intervention (no. of CT cycles)	Staging	CT regimen	Study purpose	End points	Tumor burden	Quality of cytoreduction	Median PFS (mos)	Median OS (mos)
Vergote et al. (2010) RCT: EORTC 55971	*N* = 632 PDS/ACT (6) = 310 vs. NACT (3)/IDS/ACT (3) = 322	PDS vs. NACT IIIC = 77% vs. 76% IV = 23% vs. 24%	Platinum based	Demonstrate noninferiority of NACT compared to PDS	1°: OS 2°: adverse effects, QoL, PFS	Largest preoperative tumor size, PDS vs. NACT: ≤2 cm: 6% vs. 15% ≤5 cm: 16% vs. 23% ≤10 cm: 13% vs. 13% >10 cm: 62% vs. 24% Missing data: 4% vs. 12%	PDS vs. NACT Complete: 19% vs. 51% ≤1 cm: 22% vs. 30% >1–2 cm: 12% vs. 6% >2 cm: 41% vs. 12% missing: 5% vs. 2%	12 (PDS) vs. 12 (NACT)	29 (PDS) vs. 30 (NACT)
Kehoe et al. (2015) RCT: CHORUS	*N* = 550 PDS/ACT (6) = 276 vs. NACT (3)/IDS/ACT (3) = 274	PDS vs. NACT IIA-IIIB: 11% vs. 12% IIIC = 72% vs. 71% IV = 17% vs. 15%	Platinum and taxane based	Demonstrate noninferiority of NACT compared to PDS	1°: OS 2°: PFS, QoL	Largest preoperative tumor size, PDS vs. NACT: ≤2 cm: 5% vs. 5% ≤5 cm: 21% vs. 22% ≤10 cm: 40% vs. 40% >10 cm: 32% vs. 32% Unmeasurable: 3% vs. 2%	PDS vs. NACT Complete: 17% vs. 39% ≤1 cm: 24% vs. 34% >1 cm: 59% vs. 27%	10.7 (PDS) vs. 12 (NACT) *p*=0.2923	22.6 (PDS) vs. 24.1 (NACT)
Fagotti et al. (2016) RCT: SCORPION	*N* = 110 PDS/ACT (6) = 55 vs. NACT (3)/IDS/ACT (3) = 55	PDS vs. NACT IIIC = 86% vs. 93% IV = 14% vs. 7%	Platinum and taxane based ± bevacizumab	Demonstrate superiority of NACT compared to PDS	1°: postoperative complications, PFS 2°: OS, QoL	All with high tumor load (PI score = 8–12)‡ Largest tumor size not reported	PDS vs. NACT Complete: 46% vs. 58% ≤1 cm: 46% vs. 33% >1 cm: 9% vs. 10%	15 (PDS) vs. 14 (NACT) *p*=0.72^§^	41 (PDS) vs. NR (NACT)§
Onda et al. (2016/2020) RCT: JCOG0602	*N* = 301 PDS/ACT (8) = 149 vs. NACT (4)/IDS/ACT (4) = 152	PDS vs. NACT III = 67% vs. 69% IV = 33% vs. 31%	Platinum and taxane based	Demonstrate noninferiority and reduced treatment invasiveness in NACT compared to PDS	1°: treatment invasiveness 2°: OS, safety	Tumor burden not reported	PDS vs. NACT Complete: 12% vs. 64% <1 cm: 26% vs. 18% ≥1 cm: 63% vs. 18%	15.1 (PDS) vs. 16.4 (NACT)	49 (PDS) vs. 44.3 (NACT)
Spiliotis et al. (2014) RCT: HIPEC in recurrent EOC	*N* = 120 CRS/ACT (5) = 60 Vs. CRS-HIPEC/ACT (5) = 60	Non-HIPEC vs. HIPEC III = 58% vs. 68% IV = 42% vs. 32%	HIPEC: Platinum-sensitive = cisplatin and paclitaxel Platinum-resistant = doxorubicin and paclitaxel/mitomycin	Identify the role of HIPEC	Not clearly defined	PCI, HIPEC vs. non-HIPEC: <5: 13% vs. 12% 5–9: 37% vs. 40% ≥10: 50% vs. 48%	CC-score, HIPEC vs. non-HIPEC: CC-0: 55% vs. 65% CC-1: 33% vs. 20% CC-2: 12% vs. 15%	Not reported	13.4 (non-HIPEC) vs. 26.7 (HIPEC) *p*=0.006
Van driel et al. (2018) RCT: HIPEC in primary EOC	*N* = 245 NACT (3)/IDS/ACT (3) = 123 vs. NACT (3)/IDS-HIPEC/ACT (3) = 122	All stage III	Platinum and taxane based	Assess the efficacy and safety of IDS with and without HIPEC	1°: PFS 2°: OS, adverse effects, QoL	Number of regions involved with disease¶, non-HIPEC vs. HIPEC: 0–5: 67% vs. 68% 6–8: 33% vs. 32%	Non-HIPEC vs. HIPEC Complete: 67% vs. 69% ≤0.25 cm: 20% vs. 18% >0.25–1 cm: 11% vs. 11% >1 cm: 1% vs. 0% None: 2% vs. 3%	10.7 (non-HIPEC) vs. 14.2 (HIPEC) *p*=0.003	33.9 (non-HIPEC) vs. 45.7 (HIPEC) *p*=0.02

ACT: adjuvant chemotherapy, CT: chemotherapy, EOC: eithelial ovarian cancer, IDS: interval debulking surgery, mos: months, NA: not applicable, NACT: neoadjuvant chemotherapy, OS: overall survival, PDS: primary debulking surgery, PFS: progression-free survival, PI: predictive index, QoL: quality of life, RCT: randomized controlled trial. ^*∗*^EOC includes epithelial ovarian carcinoma, fallopian tube carcinoma, or primary peritoneal carcinoma. ‡Laparoscopic predictive index as defined by Fagotti et al. §Overall and progression-free survival reported in ASCO 2018 [24]. ¶As described by Verwaal et al., Journal of Clinical Oncology, vol. 21, no, 20, pp. 3737–3743, 2003.

## Data Availability

The data used to support the findings of this study are available from the corresponding author upon request.

## Abbreviations

AEOC: Advanced epithelial ovarian, fallopian tube, and primary peritoneal cancers
ASC: Adjuvant systemic chemotherapy
CC: Completeness of cytoreduction
CR: Complete response
CRS: Cytoreductive surgery
CT: Computerized tomography
ECOG: Eastern cooperative oncology group
HIPEC: Hyperthermic intraperitoneal chemotherapy
ICU: Intensive care unit
IQR: Interquartile range
LMWH: Low-molecular weight heparin
NACT: Neoadjuvant systemic chemotherapy
OS: Overall survival
PCI: Peritoneal cancer Index
PDS: Primary debulking surgery
PFS: Progression-free survival
RECIST: Response evaluation criteria in solid tumors.
